# Prevalence of K13 mutation and Day-3 positive parasitaemia in artemisinin-resistant malaria endemic area of Cambodia: a cross-sectional study

**DOI:** 10.1186/s12936-017-2024-4

**Published:** 2017-09-13

**Authors:** Soy Ty Kheang, Siv Sovannaroth, Sovann Ek, Say Chy, Phally Chhun, Sokkieng Mao, Sokomar Nguon, Dy Soley Lek, Didier Menard, Neeraj Kak

**Affiliations:** 1 University Research Co. LLC, Phnow Penh, Cambodia; 2 National Center for Parasitology, Entomology and Malaria Control, Phnow Penh, Cambodia; 3 Pasteur Institute in Cambodia (IPC), Phnow Penh, Cambodia

**Keywords:** Malaria, Cambodia, Surveillance, Artemisinin, Case management, Artemisinin resistant malaria

## Abstract

**Background:**

The presence of artemisinin-resistant malaria parasites was confirmed in western Cambodia in 2009. In 2013, mutations in the propeller domain of the kelch protein K13 was found to be associated with artemisinin resistance. A cross-sectional study was conducted to determine the prevalence of Day-3 parasitaemia, estimate the frequency of *k13* molecular marker and assess their relationship in the context of operational research.

**Methods:**

Blood smears and filter paper blood spots were collected from febrile patients in Kravanh District, Pursat Province. The blood smears were examined by microscopy, and blood spots by a *k13* mutation assay.

**Results:**

Data from 92 patients were analysed. Only one was positive for Day-3 parasitaemia. Results of the *k13* assay were interpretable for 76 of the 92 samples. The findings were: wild type: 9 (12%), C580Y: 64 (84%), Y493H: 3 (4%). Therefore, despite the high prevalence of *k13* mutants (67/76: 88%), only 1 of the 92 patients remained blood smear positive for *Plasmodium falciparum* on Day-3.

**Conclusions:**

These preliminary findings suggest good potency of artemisinin despite the dominance of *k13* mutation in Kravanh, but the result is not necessarily representative of the western part of Cambodia. Further investigation should be made to determine if *k13* marker remains useful as a tool for tracking artemisinin resistance and predicting the trend of the efficacy of artemisinin combination therapy once the mutant alleles have been well established in the population.

## Background

Artesunate in combination with mefloquine (A+M) has been the first-line regimen for the treatment of *Plasmodium falciparum* malaria in Cambodia since 2000. Within a few years, local malaria control staff began to observe frequent recurrences of infection among patients in western Cambodia especially in Pailin Province and nearby areas. Therapeutic efficacy studies (TES) independently conducted in Pailin and across the border in Thailand’s Chanthaburi Province during 2002–2003 indicated A+M treatment failures [1, 2]. Artemisinin resistance was suspected. It was not until 2008, when a formal study began to investigate the efficacy status of artemisinin-based combination therapy (ACT) in the area. Based on clinical data, including the presence of Day-3 positive parasitaemia suggestive of poor artemisinin potency, it became widely accepted that artemisinin resistance had emerged in that area [3]. Given the entrenched mefloquine resistance in Thailand and Cambodia, the findings explained the ACT treatment failures.

The first-line therapy for falciparum malaria in Pailin was switched to atovaquone–proguanil (Malarone^®^) during 2013–2014. In nearby Pursat Province, dihydroartemisinin–piperaquine (DHA-PIP) replaced A+M in 2012 at the time when DHA-PIP was deployed countrywide. The Cambodian national therapeutics guidelines earlier adopted DHA-PIP (in principle) as the first line drug for both *P. falciparum* and *Plasmodium vivax* in 2010.

In both Pursat and Pailin, DHA-PIP efficacy also dropped significantly from 2008 to 2010 and prevalence of Day-3 positive parasitaemia increased from 25 to 45% in Pursat and from 8 to 10% in Pailin [4]. TES monitoring of DHA-PIP by the National Center for Parasitology, Entomology and Malaria Control (CNM) found that in 2012, 46% of the patients were Day-3 positive in Pursat and 15% in Tasanh District in Battambang Province, adjacent to Pailin; in 2014, 13% were Day-3 positive in Pursat and 41% in Pailin [5].

In 2013, Ariey et al. identified mutations in the propeller domain of the kelch protein K13 to be associated with artemisinin resistance as measured by Ring Stage Survival assays and delayed parasite clearance times in *P. falciparum* isolates from Cambodia [6]. Allele C580Y was found to be the most common mutant in western Cambodia. Its prevalence in Pailin increased progressively from 40% in 2001 to about 90% in 2012.

A cross-sectional study was conducted in an area of Cambodia which had artemisinin-resistant, endemic malaria to determine the prevalence of Day-3 parasitaemia, estimate the frequency of *k13* molecular marker and assess their relationship.

## Methods

The study utilized data that was collected as part of routine health service delivery. During 29 June 2014 to 18 July 2014, febrile patients presented to a local pharmacy in Kravanh District in Pursat Province were routinely screened with combo-rapid diagnostic tests (RDT) (CareStartTM HRP2/pLDH Combo test for the detection of *P. falciparum* and pan-malaria). Extra blood drops were collected for a malaria smear and onto filter paper. Patients positive for *P. falciparum* were treated with DHA-PIP according to the national treatment guidelines. The first dose was taken under the supervision of and observed by the pharmacy owner, who also provided the patients with clear explanation for consumption of the remaining two doses at home. All patients were asked again to confirm completion of DHA-PIP doses when they returned on Day-3 for clinical and parasitological follow-up. The blood smears and filter paper blood spots also allowed us to determine the prevalence of Day-3 parasitaemia, estimate the frequency of K13 molecular marker and assess their relationship. Patients’ demographic data were collected along with interview data on recent exposure to malaria. A verbal consent was obtained from every patient, which is sufficient as part of operational research in Cambodia.

An experienced malaria clinic laboratory technician equipped with microscopy set up at the pharmacy and performed parallel Giemsa microscopy cross-checks. RDT confirmed cases were included in this study. A total of 100 patients detected positive for *P. falciparum* or mixed *P. falciparum* + non-*P. falciparum* were included. Among these 100 cases, blood smears were repeated on Day-3 and examined by microscopy if parasitaemia persisted. There was no follow up beyond Day-3 and the study was not designed to assess ACT therapeutic efficacy.

All malaria smears were reviewed blindly by a highly experienced, certified microscopist from CNM, who routinely reads malaria smears for major clinical trials. For the purpose of data analysis, the CNM reference microscopist’s readings were considered final. Blood spots were submitted to the Institut Pasteur of Cambodia for *k13* mutation assay, using the Sanger sequencing method for genotyping.

## Results and discussion

In this patient group, there were 70% local residents and 30% migrants. Among them, 93% were men and 95% were adults aged 15–49 years old. All of them were exposed to malaria in the farm or the forest in the past month.

Among the 100 Day-0 blood smears, 8 were read as negative by the reference microscopist. These were excluded from data analysis. There was one false negative reading by the local microscopist; a Day-3 negative smear was reported as *P. falciparum* with very low parasite density (32/mcL) by the reference microscopist.

Data from 92 patients were analysed. Only one was positive for Day-3 parasitaemia. Results of the *k13* assay were interpretable for 76 of the 92 samples. The findings were: Wild Type: 9 (12%), C580Y: 64 (84%), Y493H: 3 (4%). Therefore, despite the high prevalence of *k13* mutants (67/76: 88%), only one of the 92 patients remained blood smear positive for *P. falciparum* on Day-3. The Day-3 positive sample was identified positive C580Y.

In Cambodia, after mefloquine withdrawal, the in vitro sensitivity to mefloquine improved along with a decrease in the prevalence of multiple *pfmdr1* copies numbers (a marker of mefloquine resistance) [7]. However, in the case of artemisinin resistance, as observed in this observation, while *k13* mutants have almost replaced the wild type in Kravanh, Day-3 positive parasitaemia is rare. The results indicate a lack of correlation between *k13* mutation and the presence of Day-3 parasitaemia. Although evidence of DHA-PIP resistance was detected in the study area in 2014, in reality the first-line treatment was still DHA-PIP in February 2016. The findings of this study supported the continuation of the use of DHA-PIP.

## Conclusions

These preliminary findings suggest good potency of artemisinin despite the dominance of *k13* mutation in Kravanh. This raises some concern over the relevance of highly prevalent *k13* mutation to artemisinin resistance. Further investigation should be made to determine if *k13* marker remains useful as a tool for tracking artemisinin resistance and predicting the trend of ACT efficacy once the mutant alleles have been well established in the population.

When interpreting the findings, there is need for caution since the data were obtained as part of an ongoing service delivery, not collected under a stringent research setting. The timing of Day-3 parasitaemia samples was not based on an exact 72-h duration from the time of administration of the first DHA-PIP dose (the Day-3 collection from patients was beyond 72 h and <96 h) and the second and third doses might not have been exactly 24 h apart. In addition, slides were primarily read by a laboratory technician from the local health centre. However, slide cross-checks by the reference microscopist showed <10% discrepancy suggesting that the local microscopist had adequate competency to serve in such a remote malaria endemic area as in Kravanh. The microscopist also detected very low-density parasitaemia in several smears that might have been missed by most field microscopists. Overall, these preliminary data are considered sufficient to warrant a comprehensive study to re-assess the relationship between *k13* mutation and artemisinin resistance in western Cambodia.

## Abbreviations

A+M: artesunate–mefloquine combination
ACT: artemisinin-based combination therapy
APCR: adequate clinical-parasitological response
CNM: National Center for Parasitology, Entomology and Malaria Control
DHA-PIP: dihydroartemisinin–piperaquine
RDT: rapid diagnostic test
TES: therapeutic efficacy study
