# Correction to: Prevalence of K13 mutation and Day-3 positive parasitaemia in artemisinin-resistant malaria endemic area of Cambodia: a cross-sectional study

**DOI:** 10.1186/s12936-017-2073-8

**Published:** 2017-10-27

**Authors:** Soy Ty Kheang, Siv Sovannaroth, Sovann Ek, Say Chy, Phally Chhun, Sokkieng Mao, Sokomar Nguon, Dy Soley Lek, Didier Menard, Neeraj Kak

**Affiliations:** 1 PMI/USAID-Cambodia Malaria Elimination Project (CMEP), University Research Co. LLC, Phnom Penh, Cambodia; 2 National Center for Parasitology, Entomology and Malaria Control, Phnow Penh, Cambodia; 3 Pasteur Institute in Cambodia (IPC), Phnow Penh, Cambodia

## Correction to: Malar J (2017) 16:372 DOI 10.1186/s12936-017-2024-4

After publication of the article [1], it has been brought to our attention that the funding acknowledgements for this article are incomplete. The authors would like to also include the following:

“The URC staff participating in this study were funded by the President's Malaria Initiative/United States Agency for International Development (PMI/USAID) under AID-486-A-12-00001—Control and Prevention of Malaria (CAP-Malaria) and AID-442-C-17-00001—Cambodia Malaria Elimination Project (CMEP) projects.”
